# Two new species of *Taiwanocantharis* from Yunnan Province, China, with a worldwide key to the genus (Coleoptera, Cantharidae)

**DOI:** 10.3897/BDJ.14.e192348

**Published:** 2026-07-27

**Authors:** Ruobing Hua, Haoyu Liu, Xingke Yang, Yuxia Yang

**Affiliations:** 1 Key Laboratory of Zoological Systematics and Application, College of Life Sciences, Hebei University, Baoding, China Key Laboratory of Zoological Systematics and Application, College of Life Sciences, Hebei University Baoding China; 2 Hebei Basic Science Center for Biotic Interaction, Hebei University, Baoding, China Hebei Basic Science Center for Biotic Interaction, Hebei University Baoding China; 3 Key Laboratory of Zoological Systematics and Evolution, Institute of Zoology, Chinese Academy of Sciences, Beijing, China Key Laboratory of Zoological Systematics and Evolution, Institute of Zoology, Chinese Academy of Sciences Beijing China

**Keywords:** alpha taxonomy, Oriental Region, soldier beetles

## Abstract

**Background:**

*Taiwanocantharis* Wittmer, 1984 is a genus of soldier beetles that are remarkable for their metallic green or blue elytra, medium-sized body, subrounded pronotum and usually appendiculate outer tarsal claws in both sexes. Currently, the genus comprises 19 species distributed across the Indochina Peninsula and the Himalayan Region.

**New information:**

Two new species of *Taiwanocantharis* have been discovered and described under the names of T. flavipes **sp. nov**. and T. liangi **sp. nov**. (China, Yunnan). Both species are illustrated with habitus of both sexes, aedeagus, as well as abdominal sternite VIII and reproductive systems of the female. An updated key to the genus is provided.

## Introduction

*Taiwanocantharis* Wittmer, 1984 is a remarkable genus of soldier beetles particularly characterised by their metallic green or blue elytra, medium-sized body, subrounded pronotum and usually appendiculate outer tarsal claws in both sexes (Švihla 2011). To date, this genus comprises a total of 19 species distributed across the Indochina Peninsula and the Himalayan Region, with most species described by Wittmer (1984), Wittmer (1997a), Wittmer (1997b), Švihla (2004), Švihla (2005), Yang and Yang (2014) and Hua et al. (2026).

In this study, we describe two unknown species discovered in Yunnan, China. These findings will enhance the taxonomic knowledge of *Taiwanocantharis* and provide essential reference data for future phylogenetic analyses for this genus and its allied taxa.

## Materials and methods

The studied specimens are deposited in the Institute of Zoology, Chinese Academy of Sciences, Beijing, China (IZAS) and Museum of Hebei University, Baoding, China (MHBU).

The genitalia of both sexes and the abdominal sternites VIII of females were dissected and cleared in 10% sodium hydroxide (NaOH) solution. Subsequently, male genitalia were soaked in a warm 30% NaOH solution for an appropriate duration (generally between 5 to 15 minutes, depending on their condition and size). Following this, the male genitalia were cleaned and female genitalia were dyed with haematoxylin. Habitus photos were taken using a Canon EOS 80D digital camera, while images of the genitalia were captured via a Leica M205A stereomicroscope. Finally, the genitalia were permanently perserved in the glycerol.

Morphological comparisons were performed under a stereomicroscope. Body length was measured from the clypeus to the elytral apex and width at the humeral widest point. For male genitalia, the relative lengths of the dorsal plate and ventral process were assessed in lateral view to determine whether one extended distally beyond the tip of the other (longer than) or both terminated at approximately the same horizontal level (subequal in length). Images were processed with Helicon Focus 7 and Adobe Photoshop CS5.

Complete label data for type specimens were provided. Quotation marks were used to denote original English-language labels, whereas all labels originally written in Chinese were transliterated into English.

## Taxon treatments

### Taiwanocantharis

Wittmer, 1984

57626ACE-7F70-5250-AE02-A64AEA104EAA

Cantharis (Taiwanocantharis) Wittmer, 1984 - Wittmer (1984): 147.
Taiwanocantharis Wittmer, 1984 - Švihla (2011): 4.
Cantharis (Taiwanocantharis) tripunctata Wittmer, 1984

#### Diagnosis

Body middle-sized. Head black, pronotum yellow, often with black markings, elytra metallic green or blue. Antennae filiform, antennomeres IV-XI with narrow, smooth, longitudinal or oval grooves along outer margins. Pronotum nearly circular, with posterior angles more or less protruding laterally. Legs usually with all outer claws sharply appendiculate in both sexes. Aedeagus with conjoint dorsal plate of parameres and a pair of well-developed laterophyses.

### Taiwanocantharis flavipes

Y. Yang, Hua & X. Yang sp. nov.

C499A392-7A79-5835-BCE1-1E09C9722350

3F0E0FCA-2AAE-4CAA-AE91-7CA4CDF9DF50

#### Materials

- **Type status:** Holotype. **Occurrence:** recordedBy: Liang H. B.; Hu P.; sex: 1 male; lifeStage: adult; occurrenceID: D9033DF5-CB34-5439-8AE6-5D2644B2FA27; **Location:** country: China; stateProvince: Yunnan; county: Tengchong; locality: Jietou town, Shaba, Chahe, Vegetation; verbatimElevation: 1850 m; **Event:** year: 2006; month: 5; day: 25; **Record Level:** institutionID: Institute of Zoology, Chinese Academy of Sciences; institutionCode: IZAS
- **Type status:** Paratype. **Occurrence:** recordedBy: 采集人: Yuxia Yang 杨玉霞; sex: 1 female; lifeStage: adult; occurrenceID: DFFF95DA-5DB7-5F8C-8693-995333E5843C; **Location:** country: China中国; stateProvince: Yunnan云南; county: Baoshan保山; locality: Gaoligong Mts.高黎贡山; verbatimElevation: 海拔2250 m; **Event:** year: 2011; month: 6; day: 21-24; **Record Level:** institutionID: Museum of Hebei University, Baoding, China; institutionCode: MHBU 河北大学博物馆

#### Description

Body length 10.8‒12.4 mm (10.8 mm in holotype); width 2.1‒3.4 mm (2.1 mm in holotype).

**Male** (Fig. 1a): Body yellow, head black, antennomeres III‒XI pale brown, pronotum yellow, with a pair of large subtrapezoidal black markings in centre and two pairs of small prebasal black markings on both sides, elytra green, with strongly metallic lustere. Body densely covered with light yellow recumbent pubescence, of which they are feebly sparser on head.

Head rounded (dorsal view), densely punctate on surface; eyes slightly protruding, head width at the eyes nearly as wide as pronotum; antennae filiform, extending to the middle of elytra when inclined, antennomeres II shorter and about 1.3 times longer than I, III approximately 1.5 times as long as II, V longer, XI feebly longer than X, IV‒XI each with a narrow, smooth longitudinal to oval groove near the middle of the outer margin, of which those on VI and VII are the longest.

Pronotum 1.2 times wider than long, anterior margin feebly arcuate, lateral margins rounded, posterior margin nearly straight, anterior angles widely rounded, posterior angles rounded, not protruding, disc distinctly convex on postero-lateral parts, surface lustrous, slightly largely and sparsely punctate.

Elytra nearly parallel-sided, 3.2 times longer than humeral width, 4.8 times longer than pronotum, surface coarsely punctate. Each outer tarsal claw sharply appendiculate, while inner claws simple.

Aedeagus (Fig. 2a, b, c): ventral process of each paramere slender and nearly straight, feebly expanded apically and rounded at apices in ventral view (Fig. 2b); conjoint dorsal plate of parameres longer than ventral processes (Fig. 2c), with simple and widely triangular lateroapical angles (Fig. 2c), apical margin roundly emarginate in middle (Fig. 2a); laterophyses reaching the bottom of middle emargination, separated bilaterally, feebly expanded apically and truncate at apices (Fig. 2a), which beak-like at lateroapical angles (Fig. 2b).

**Female** (Fig. 1b). Similar to male, but eyes smaller, terminal maxillary palpomeres shorter, nearly widest in middle, antennae shorter, extending to basal third length of elytra, antennomeres IV–XI without any grooves, pronotum slightly darkened along lateral margins, elytra with lateral margins slightly diverging posteriorly, all tarsal claws simple. Abdominal sternite VIII (Fig. 3a) largely and rectangularly emarginate in middle of posterior margin, each side with a triangular protuberance at bottom of the middle emargination. Internal organ of reproductive system (Fig. 3c): diverticulum moderately long, thin and spiral tube-shaped, almost evenly thin along whole length; spermathecal duct distinctly shorter and feebly stouter than diverticulum; spermatheca thin and spiral tube-shaped, about 2.5 times as long as diverticulum; accessory gland evenly thin and nearly 1/3 length of spermatheca.

#### Diagnosis

This species is similar to *T. dedicata* (Švihla, 2005) in the general shape of aedeagus, but can be easily differentiated from *T. dedicata* by the uniformly yellow legs (Fig. 1a, b), while black in *T. dedicata*; aedeagus with laterophyses beak-like at lateroapical angles (Fig. 2a), while triangular in *T. dedicata* (Švihla 2005: fig. 35); ventral process of each paramere feeble.

#### Etymology

The specific name is derived from the Latin *flavus* (yellow) and *pes* (foot), referring to its uniformly yellow legs.

#### Distribution

China (Yunnan).

### Taiwanocantharis liangi

Y. Yang, Hua & X. Yang sp. nov.

323264ED-1EDC-5A7D-9C69-CBD6D0128457

481AD8CB-8182-419F-B9A0-7F80B88B5E00

#### Materials

- **Type status:** Holotype. **Occurrence:** recordedBy: Liang H. B.; Hu P.; sex: 1 male; lifeStage: adult; occurrenceID: E0921BD2-0063-538A-A988-12E7C341227A; **Location:** country: China; stateProvince: Yunnan; county: Tengchong; locality: Mingguang, Zizhi; **Event:** year: 2006; month: 5; day: 23; **Record Level:** institutionCode: Institute of Zoology, Chinese Academy of Sciences, Beijing, China; collectionCode: IZAS
- **Type status:** Paratype. **Occurrence:** recordedBy: Hao Huang; sex: 1 male; lifeStage: adult; occurrenceID: 556CB462-BE28-5C69-8253-26588831563A; **Location:** country: China; stateProvince: Yunnan; county: Tengchong; locality: Datang village; **Event:** year: 2005; month: 5; day: 12; **Record Level:** institutionID: Institute of Zoology, Chinese Academy of Sciences, Beijing, China; institutionCode: IZAS
- **Type status:** Paratype. **Occurrence:** recordedBy:  Liang H. B.; Liu Z. C.; sex: 1 female; lifeStage: adult; occurrenceID: 767A21CC-75BE-5EF4-9FE5-600E6BE2671C; **Location:** country: China; stateProvince: Yunnan; county: Tengchong; locality: Mingguang, Houqiao, Guyong Forestry; **Event:** year: 2006; month: 5; day: 27; **Record Level:** institutionID: Institute of Zoology, Chinese Academy of Sciences, Beijing, China; institutionCode: IZAS
- **Type status:** Paratype. **Occurrence:** recordedBy: Liang H. B.; Hu P.; sex: 1 female; lifeStage: adult; occurrenceID: 2B420E89-9F88-5066-AFED-6D2F55FF2631; **Location:** country: China; stateProvince: Yunnan; county: Tengchong; locality: Mingguang, Zizhi; **Event:** year: 2006; month: 5; day: 23; **Record Level:** institutionID: Institute of Zoology, Chinese Academy of Sciences, Beijing, China; institutionCode: IZAS

#### Description

Body length 9.6‒10.3 mm (9.6 mm in holotype), width at humeri 2.3‒2.7 mm (2.3 mm in holotype).

**Male** (Fig. 1c): Body brownish-yellow, head black, antennae brown, antennomeres I‒II yellowish-orange ventrally, pronotum yellow, with a large black marking in centre of disc, elytra green, with strongly metallic lustre, legs slightly darkened at apical parts of femora, tibiae and tarsi. Body densely covered with light yellow recumbent pubescence, of which they are feebly sparser on head.

Head rounded, densely punctate on surface; eyes moderately protruding, head width across eyes narrower than pronotum; antennae filiform, extending to the middle of elytra when inclined, antennomeres II shortest and about half as long as I, III third longer than II, antennomere V is the longest segment, XI feebly longer than X. IV‒XI each with a narrow, smooth longitudinal to oval groove near the middle of the outer margin, with those on VI and VII being the longest amongst these grooves.

Pronotum 1.2 times wider than long, anterior margin moderately arcuate, lateral margins rounded, posterior margin nearly straight, anterior angles widely rounded, posterior angles hardly protruding, disc distinctly convex on postero-lateral parts, surface finely punctate.

Elytra nearly parallel-sided, approximately 3.1 times longer than humeral width, 4.5 times longer than pronotum, surface nearly lustrous at anterior third parts, coarsely punctate on the rest. Each outer tarsal claw triangularly appendiculate, while inner claws simple.

Aedeagus (Fig. 2d, e, f): ventral process of each paramere slender and slightly bent inwards, feebly narrowed apically and acute at apices in ventral view (Fig. 2e); conjoint dorsal plate of parameres longer than ventral processes (Fig. 2f), with moderately developed and incurved lateroapical angles (Fig. 2e), apical margin roundly emarginate in middle (Fig. 2d); laterophyses extruding over the bottom of middle emargination, separated bilaterally and curved inwards dorsally around the median lobe, with apices rounded and directing apically (Fig. 2d).

**Female** (Fig. 1d): Similar to male, but body larger, eyes less protruding, antennae shorter, extending to basal third length of elytra when inclined, middle antennomeres lacking grooves, pronotum wider and 1.3 times wider than long, feebly convex on disc, all tarsal claws simple. Abdominal sternite VIII (Fig. 3b) deeply and triangularly emarginated in the middle of posterior margin, with a pair of rounded protuberances at the lateral angles of the middle emargination. Internal organ of reproductive system (Fig. 3d): diverticulum moderately long, thin and spiral tube-shaped, progressively thinning apically; spermathecal duct shorter and feebly stouter than diverticulum; spermatheca thin and spiral tube-shaped, longer than diverticulum; accessory gland evenly thin and shorter than spermatheca.

#### Diagnosis

This species is similar to *T. metallipennis* (Wittmer, 1997b) in the general shape of aedeagus, but can be easily distinguished from the latter by a combination of the following characteristics: pronotum with the black marking not extending to the anterior or posterior margins (Fig. 1c, d), while *T. metallipennis* with the black marking extending to both anterior and posterior margins; aedeagus: conjoint dorsal plate of parameres distinctly longer than ventral processes (Fig. 2f), with shallower middle emargination at apical margin (Fig. 2d), while in *T. metallipennis*, conjoint dorsal plate of parameres nearly as long as ventral processes, with deeper middle emargination at apical margin (Wittmer 1997b: fig. 134); abdominal sternite VIII of female with a pair of rounded protuberances beside middle emargination of the posterior margin (Fig. 3b), while the protuberances acute at apices in *T. metallipennis* (Wittmer 1997b: fig. 135).

#### Etymology

The species is named after one of its types’ collector, Dr. Hongbin Liang (IZAS).

#### Distribution

China (Yunnan)

## Identification Keys

### A key to the worldwide species of *Taiwanocantharis*

**Table:** 

1	Elytra slender, ca. 4.0 times as long as humeral width	2
–	Elytra stouter, at most 3.2 times longer than humeral width	3
2	Pronotum uniformly yellow, without any black markings on disc; legs uniformly black brown	***T. pallidithorax* (Wittmer, 1984)**
–	Pronotum yellow, with a large and a pair of small black markings on disc; legs yellow, tibiae black dorsally, tarsi unifromly black	***T. tripunctata* (Wittmer, 1984)**
3	Pronotum with a single large black marking in the centre of disc (e.g.Fig. 1c, d)	4
–	Pronotum with a pair of moderately large marking in the centre and several smaller prebasal dark markings laterally on disc, sometimes the markings more or less conjoint (e.g. Fig. 1a, b)	12
4	Pronotum dark along lateral margins	5
–	Pronotum uniformly yellow along lateral margins (e.g. Fig. 1c, d; Yang and Yang (2014): figs. 1 and 2)	6
5	Elytra dark green, weakly shining; aedeagus: conjoint dorsal plate of parameres roundly emarginate in middle of apical margin, with strongly developed lateroapical teeth curved inwards (Wittmer 1997a: fig. 10)	***T. satoi* (Wittmer, 1997)**
–	Elytra bright green or blue, strongly shining; aedeagus: conjoint dorsal plate of parameres triangularly emarginate in middle of apical margin, without any lateroapical teeth (Hua et al. 2026: fig. 2D)	***T. marginalis* Y. Yang & Hua, 2026**
6	Pronotum with the black making confined to the centre, not extending to anterior or posterior margins (Fig. 1c, d)	7
–	Pronotum with the black marking extending to anterior and posterior margins (e.g. Yang and Yang (2014): figs. 1, 2 and 3)	9
7	Pronotum nearly as wide as long; aedeagus: conjoint dorsal plate of parameres protuberant and tapered apically (Švihla 2004: fig. 55)	***T. drahuska* (Švihla, 2004)**
–	Pronotum distinctly wider than long; aedeagus: conjoint dorsal plate of parameres emarginate in middle or protuberant, but truncate at apical margin	8
8	Legs mostly yellow (Fig. 1c, d); aedeagus: conjoint dorsal plate of parameres emarginate in middle of apical margin (Fig. 2d)	***T. liangi* Y. Yang, Hua & X. Yang, sp. nov**.
–	Legs mostly dark brown; aedeagus: conjoint dorsal plate of parameres protuberant, but truncate at apical margin (Wittmer 1997b: fig. 148)	***T. thibetana* (Gorham, 1889)**
9	Aedeagus: conjoint dorsal plate of parameres largely emarginate in middle of apical margin, with moderately developed lateroapical teeth; laterophyses rounded at apices, directing inner-apically (Wittmer 1997b: fig. 134)	***T. metallipennis* (Wittmer, 1997)**
–	Aedeagus: conjoint dorsal plate of parameres less emarginate in middle of apical margin, with strongly developed lateroapical teeth; laterophyses rectangular at apices, directing inwards (Yang and Yang 2014: figs. 6, 10 and 14)	10
10	Aedeagus: conjoint dorsal plate of parameres hardly emarginate in middle of apical margin, without any teeth on preapical median part of inner surface (Yang and Yang 2014: fig. 14)	***T. adentata* (Y. Yang & X. Yang, 2014)**
–	Aedeagus: conjoint dorsal plate of parameres distinctly emarginate in middle of apical margin, with a pair of small triangular teeth on pre-apical median part of inner surface (Yang and Yang 2014: figs. 6 and 10)	11
11	Aedeagus: conjoint dorsal plate of parameres longer than ventral processes (Yang and Yang 2014: fig. 6)	***T***. ***thibetanomima* (Wittmer, 1997)**
–	Aedeagus: conjoint dorsal plate of parameres as long as ventral processes (Yang and Yang 2014: fig. 10)	***T. wittmeri* Y. Yang & X. Yang, 2014**
12	Body smaller, 7.0–8.0 mm in length; aedeagus: conjoint dorsal plate of parameres feebly protuberant in middle of apical margin; laterophyses next to each other in middle (Kopetz 2008: figs 1-2)	***T. chumbiensis* (Champion, 1926)**
–	Body larger, longer than 8.0 mm; aedeagus: conjoint dorsal plate of parameres emarginate or protuberant in middle of apical margin; laterophyses separated from each other on both sides (e.g. Fig. 2a; Švihla (2005): fig. 35)	13
13	Aedeagus: conjoint dorsal plate of parameres protuberant and rounded at apical margin (Wittmer 1989: fig. 2)	***T. shergaoensis* (Wittmer, 1989)**
–	Aedeagus: conjoint dorsal plate of parameres more or less emarginate in middle of apical margin (e.g. Fig. 2a; Wittmer (1997b): fig. 133; Yang and Yang (2014): figs. 10 and 18)	14
14	Aedeagus: conjoint dorsal plate of parameres with a pair of protuberances along middle emargination of apical margin (Yang and Yang 2014: fig. 18)	***T. parasatoi* Y. Yang & X. Yang, 2014**
–	Aedeagus: conjoint dorsal plate of parameres without any protuberances along middle emargination of apical margin	15
15	Aedeagus: ventral process of each paramere gradually narrowed apically almost even in width (e.g. Fig. 2b; Wittmer (1989): figs. 1 and 5; Švihla (2005): fig. 35)	16
–	Aedeagus: ventral process of each paramre abruptly narrowed apically (e.g. Wittmer (1989): figs. 2 and 3; Wittmer (1997b): fig. 136)	20
16	Aedeagus: conjoint dorsal plate of parameres roundly emarginate in middle of apical margin (Wittmer 1989: fig. 1)	17
–	Aedeagus: conjoint dorsal plate of parameres triangularly emarginate in middle of apical margin (Wittmer 1989: fig. 5)	19
17	Aedeagus: laterophyses rounded at lateroapical angles (Wittmer 1989: fig. 1)	***T. quinquenotatithorax* (Pic, 1914)**
–	Aedeagus: laterophyses acute at lateroapical angles	18
18	Legs uniformly yellow; aedeagus: laterophyses beak-like at lateroapical angles (Fig. 2b)	***T. flavipes* Y. Yang, Hua & X. Yang, sp. nov**.
–	Legs uniformly black; aedeagus: laterophyses triangular at lateroapical angles (Švihla 2005: fig. 35)	***T. dedicata* (Švihla, 2005)**
19	Aedeagus: conjoint dorsal plate of parameres largely emarginate in middle of apical margin, laterophyses nearly straight and truncated at apices (Wittmer 1989: fig. 5)	***T. malaisei* (Wittmer, 1989)**
–	Aedeagus: conjoint dorsal plate of parameres feebly emarginate in middle of apical margin, laterophyses incurved and rounded at apices (Wittmer 1997b: fig. 133)	***T. inthanonensis* (Wittmer, 1997)**
20	Aedeagus: conjoint dorsal plate of parameres feebly emarginate in middle of apical margin, laterophyses acute at lateroapical angles (Wittmer 1989: fig. 3)	***T. kambaitiensis* (Wittmer, 1989)**
–	Aedeagus: conjoint dorsal plate of parameres largely emarginate in middle of apical margin, laterophyses rounded at lateroapical angles (Wittmer 1989: fig. 4)	***T. seinghukuensis* (Wittmer, 1989)**

## Supplementary Material

XML Treatment for Taiwanocantharis

XML Treatment for Taiwanocantharis flavipes

XML Treatment for Taiwanocantharis liangi

## Figures and Tables

**Figure 1a.**
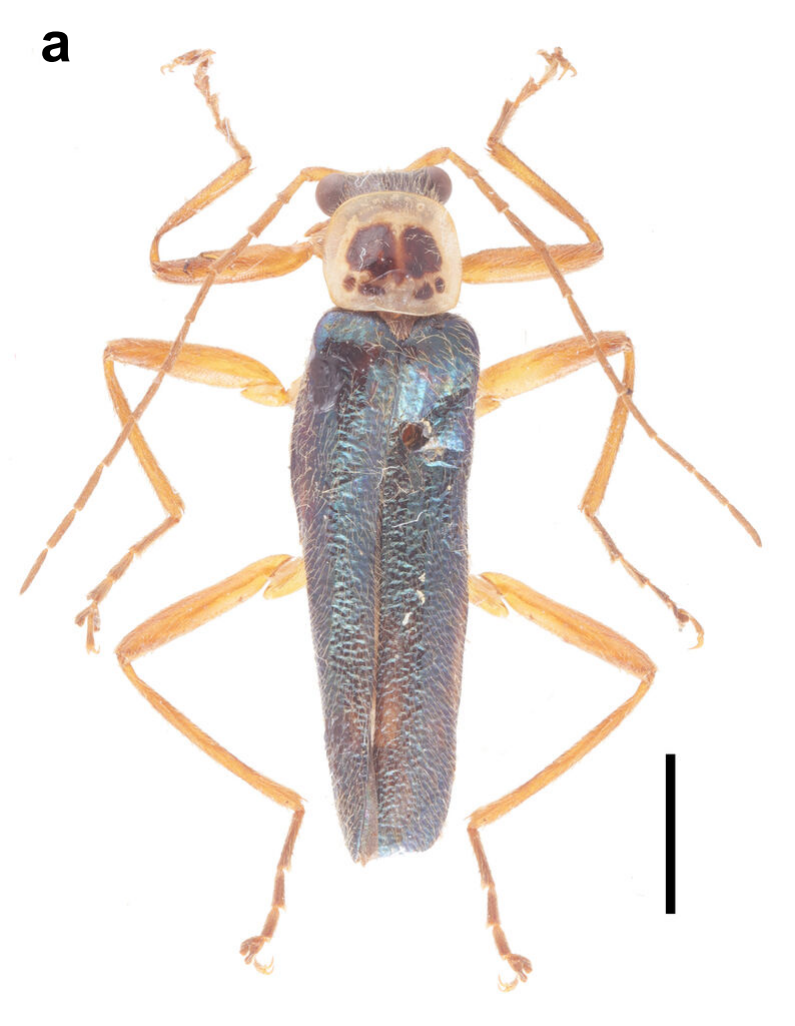
Taiwanocantharis flavipes sp. nov., male;

**Figure 1b.**
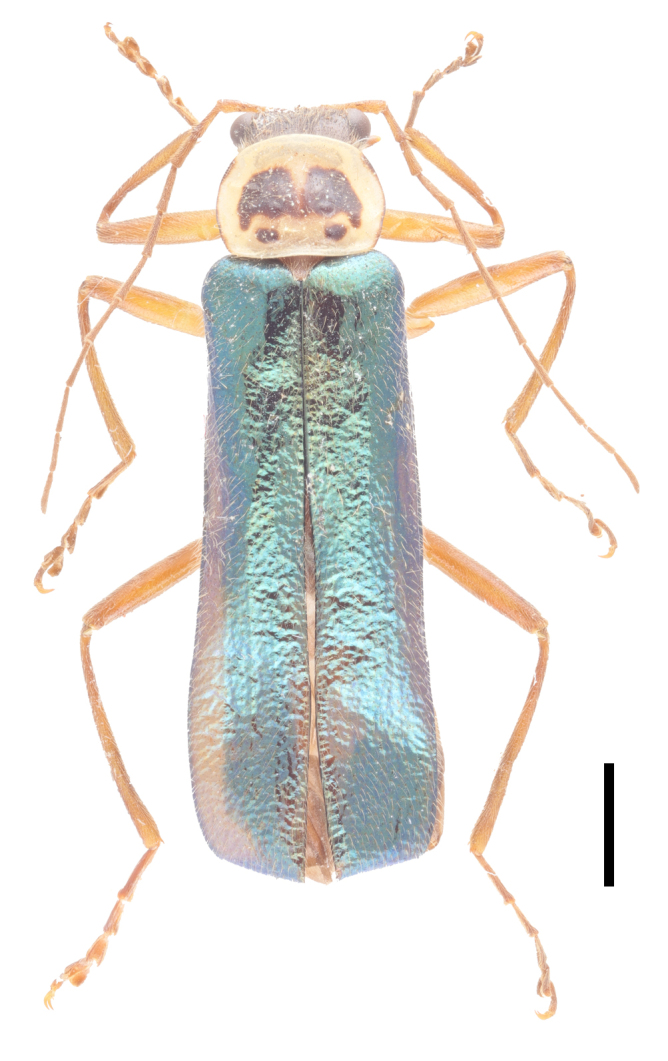
Taiwanocantharis flavipes sp. nov., female;

**Figure 1c.**
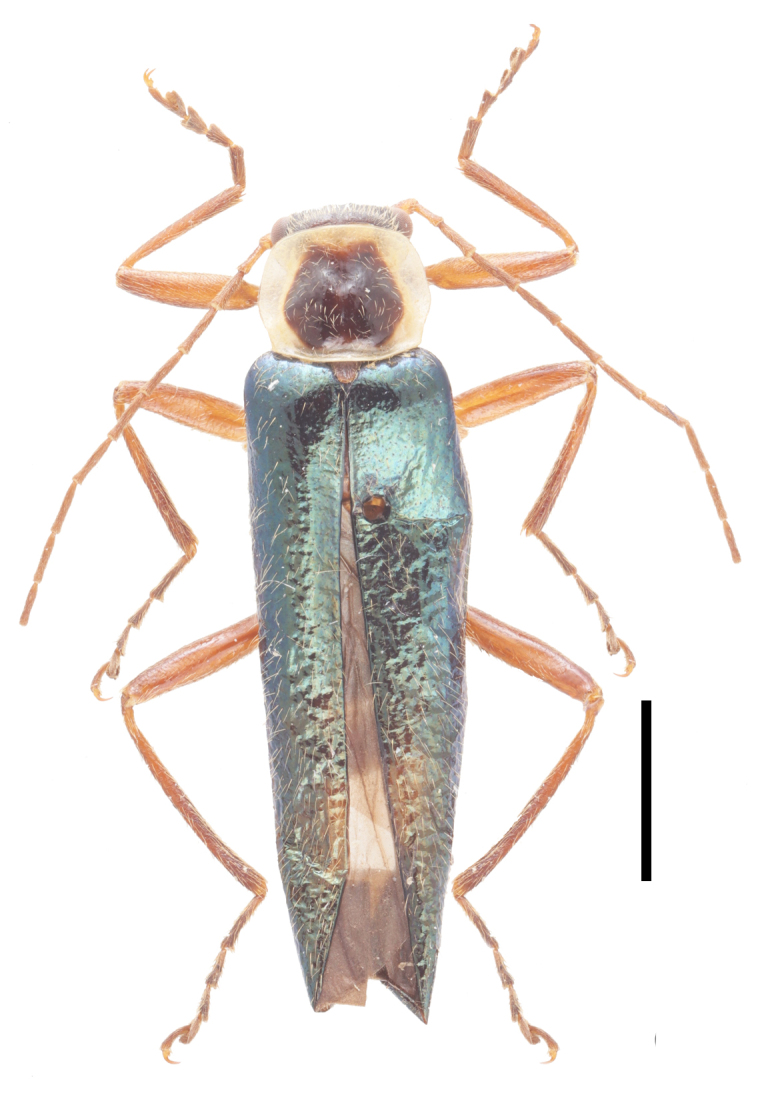
Taiwanocantharis liangi sp. nov., male;

**Figure 1d.**
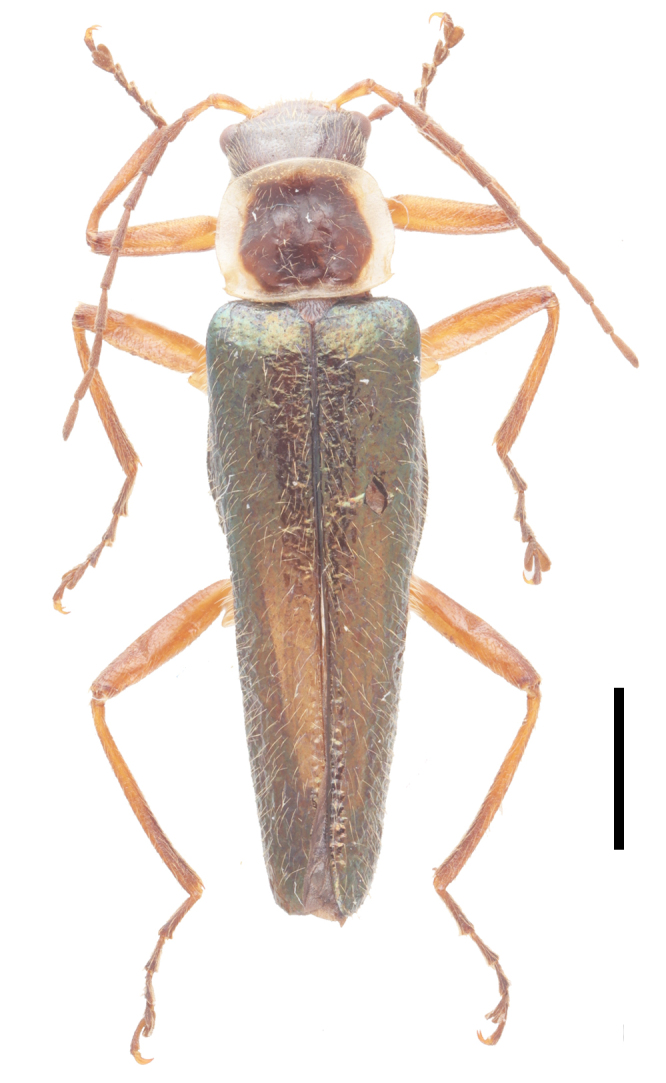
Taiwanocantharis liangi sp. nov., female.

**Figure 2a.**
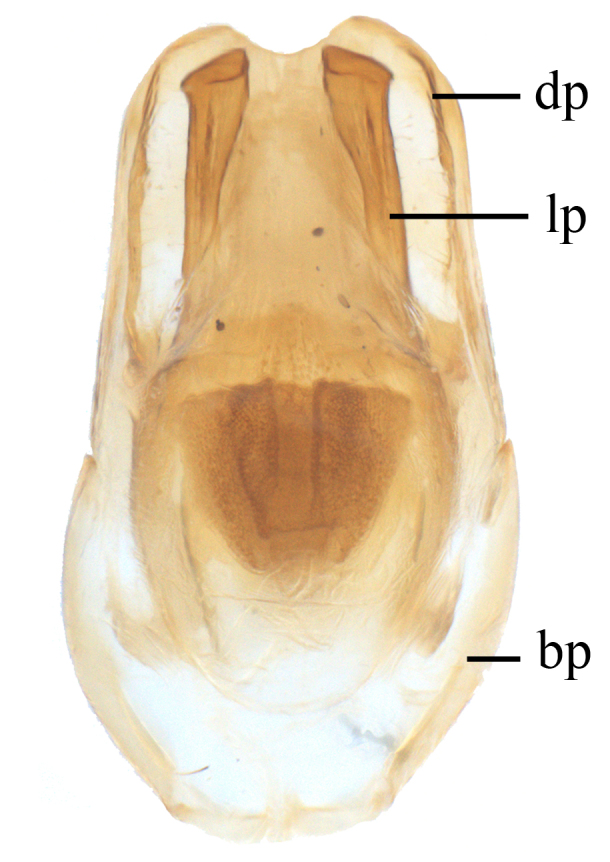
Taiwanocantharis flavipes sp. nov., dorsal view;

**Figure 2b.**
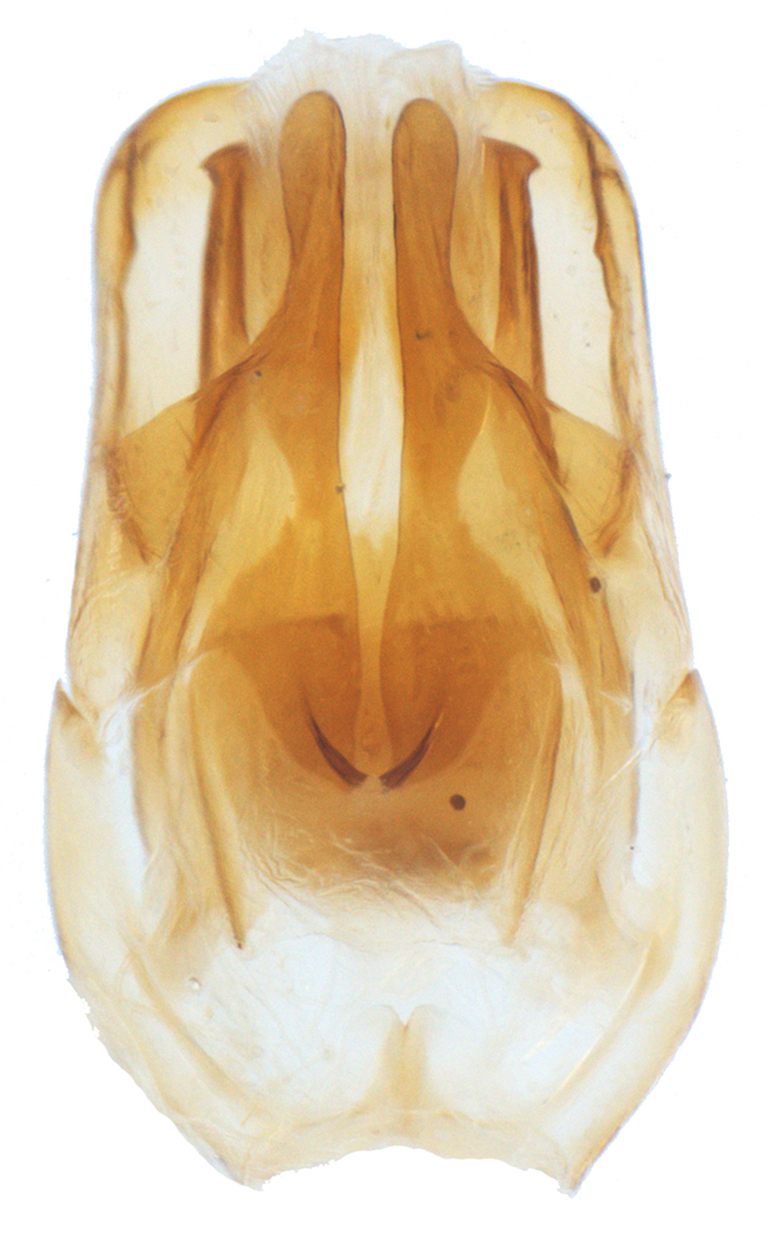
Taiwanocantharis flavipes sp. nov., ventral view;

**Figure 2c.**
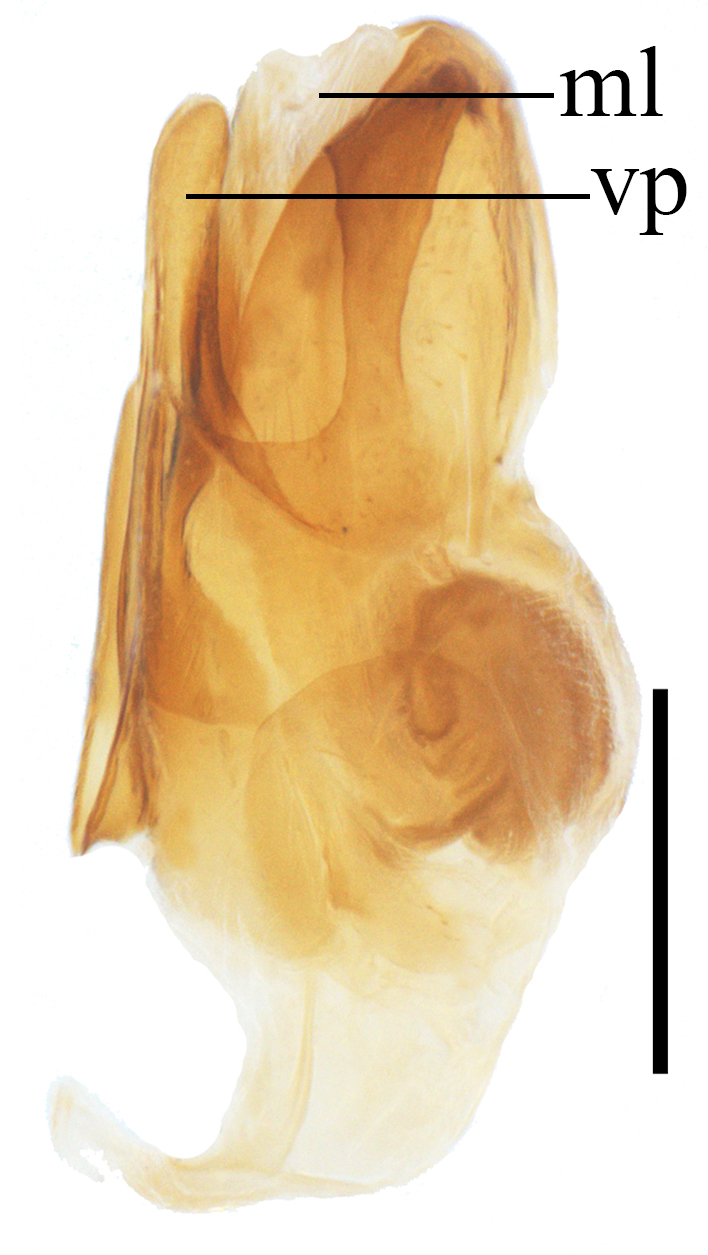
Taiwanocantharis flavipes sp. nov., lateral view;

**Figure 2d.**
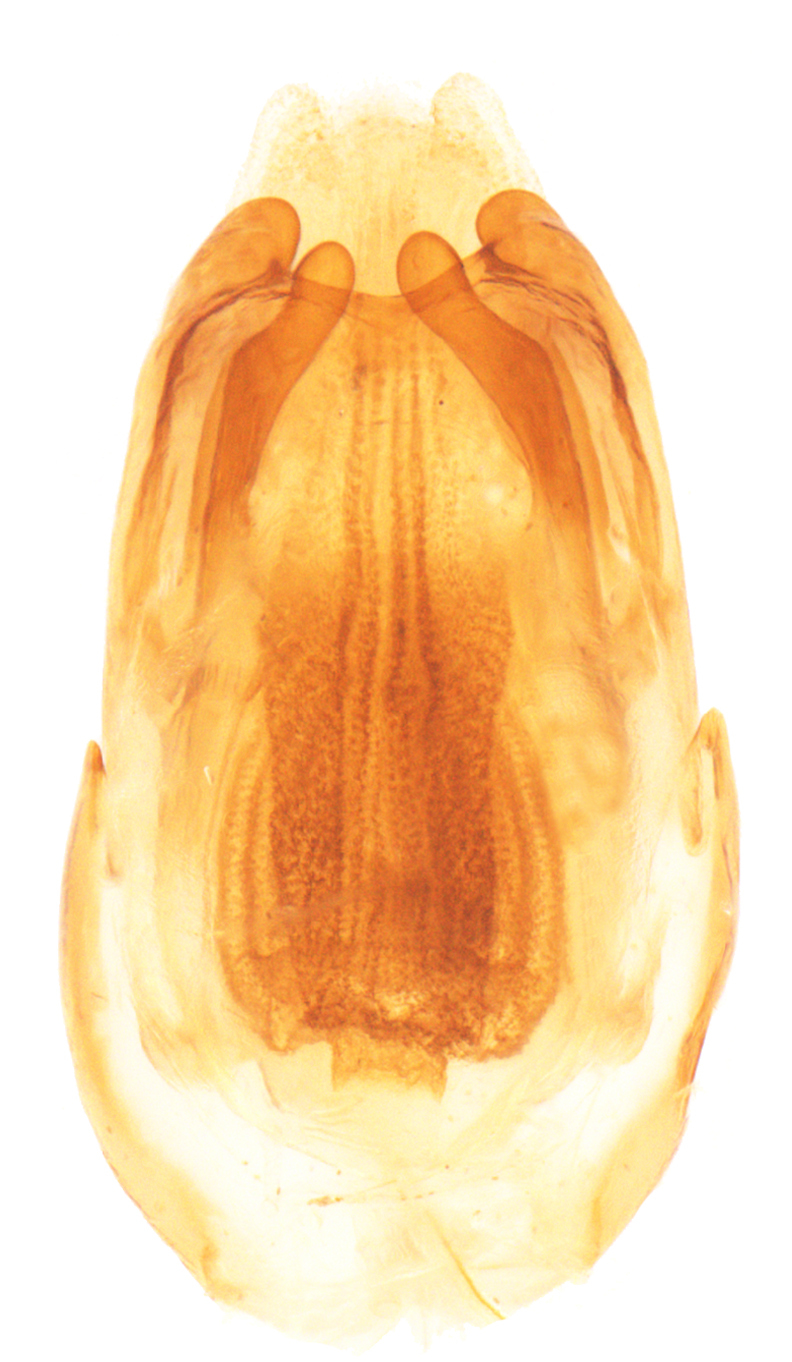
T. liangi sp. nov., dorsal view;

**Figure 2e.**
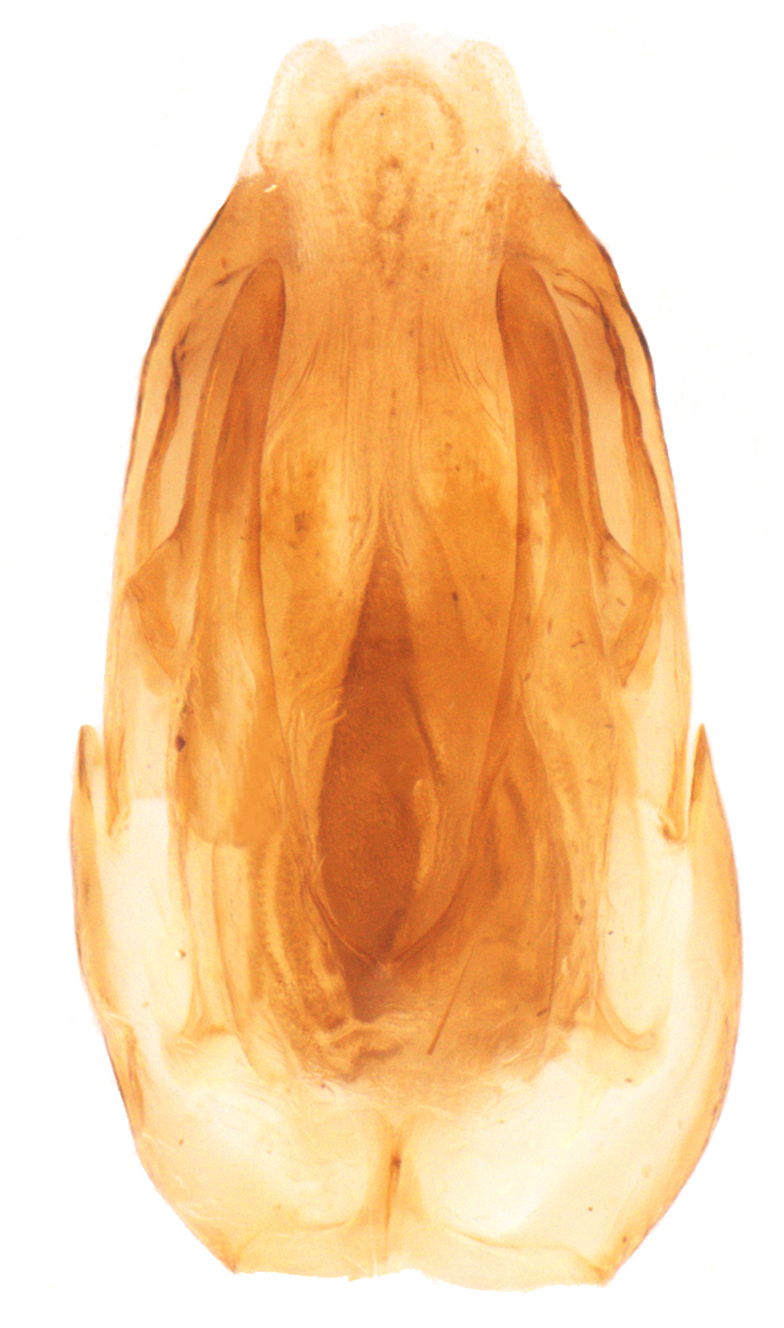
T. liangi sp. nov., ventral view;

**Figure 2f.**
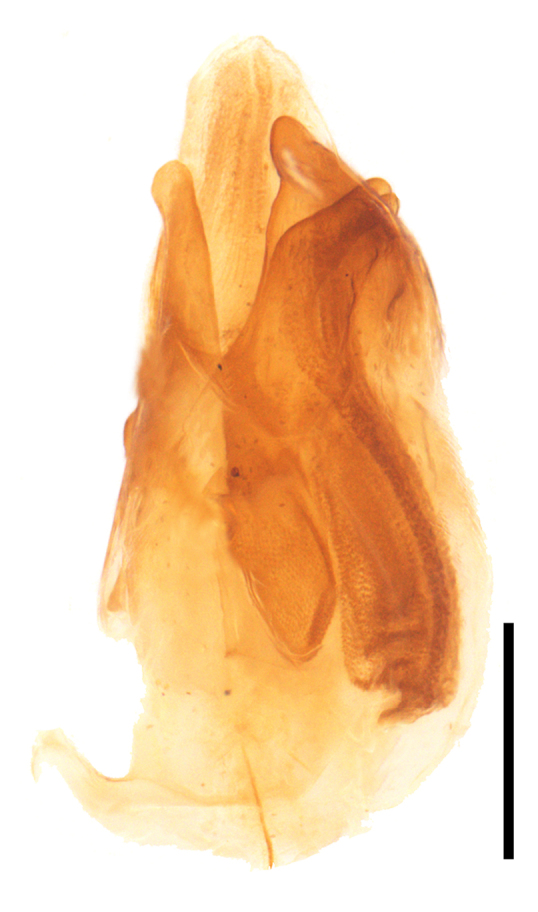
T. liangi sp. nov., lateral view.

**Figure 3a.**
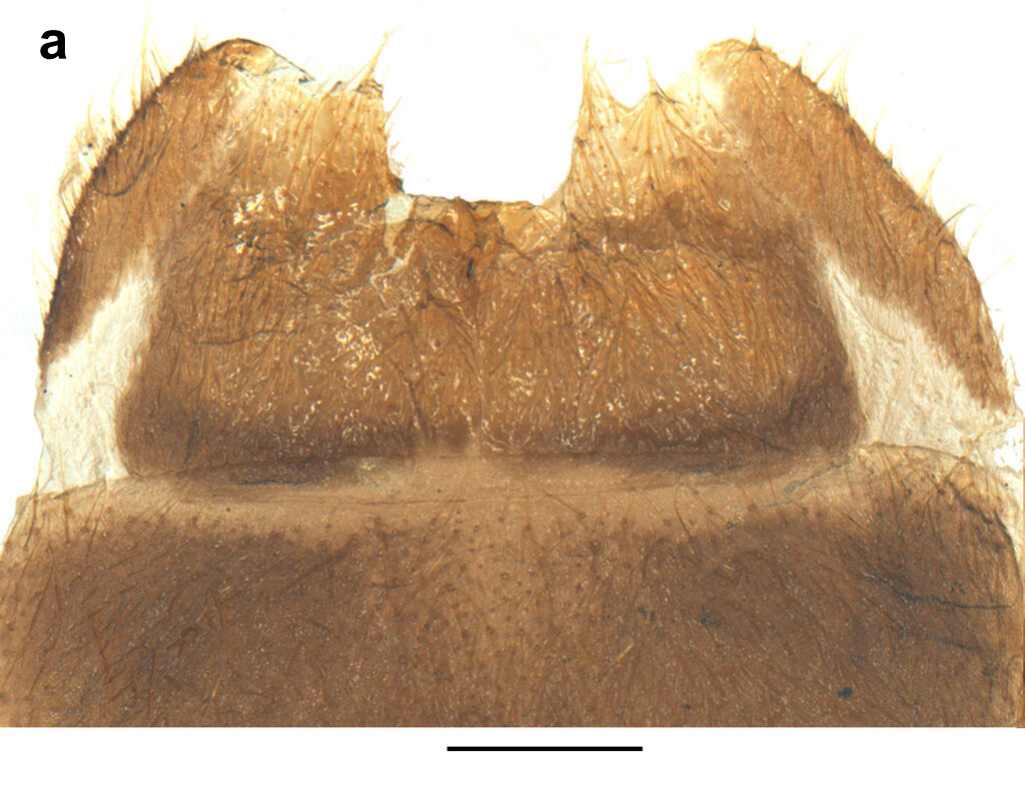
Taiwanocantharis flavipes sp. nov., abdominal sternite VIII of female, ventral view;

**Figure 3b.**
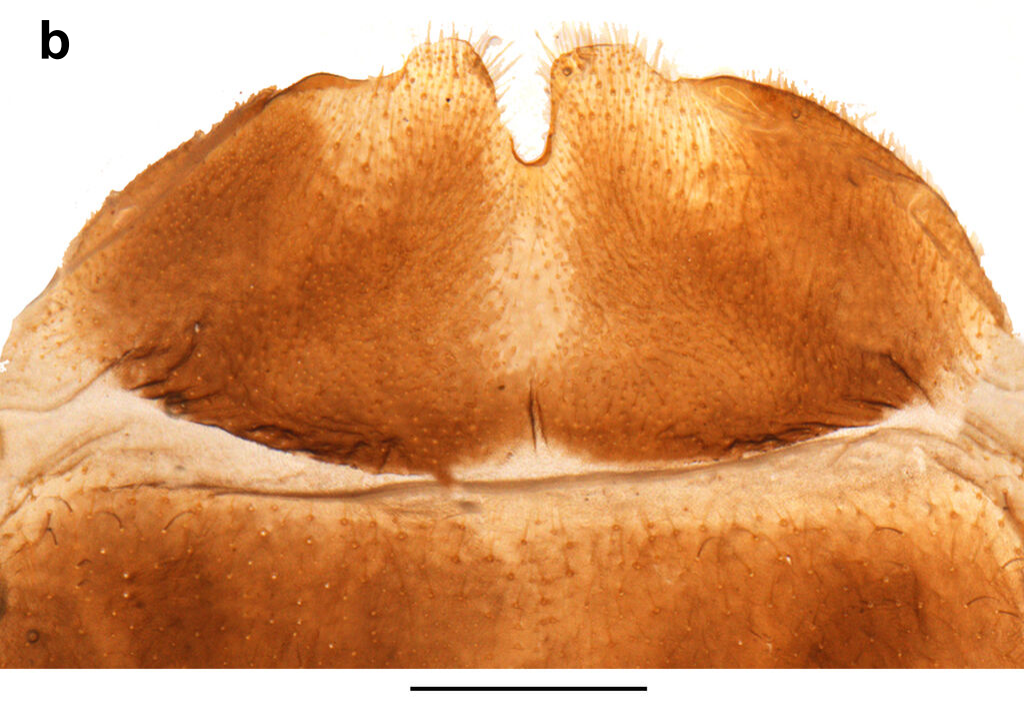
T. liangi sp. nov., abdominal sternite VIII of female, ventral view;

**Figure 3c.**
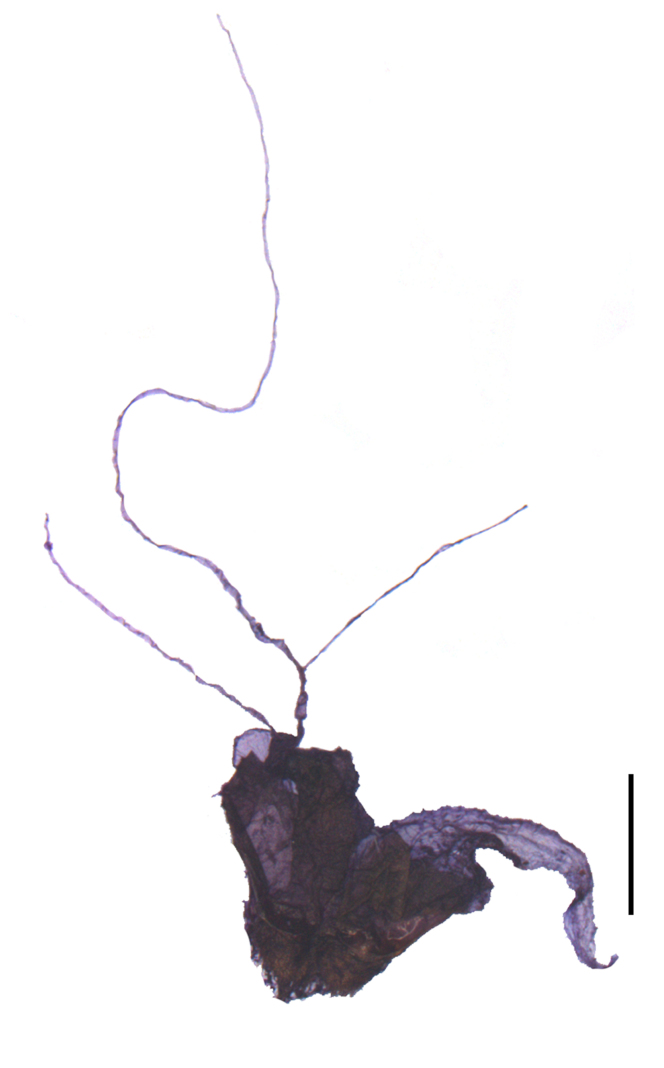
Taiwanocantharis flavipes sp. nov., internal organ of female reproductive system, lateral view;

**Figure 3d.**
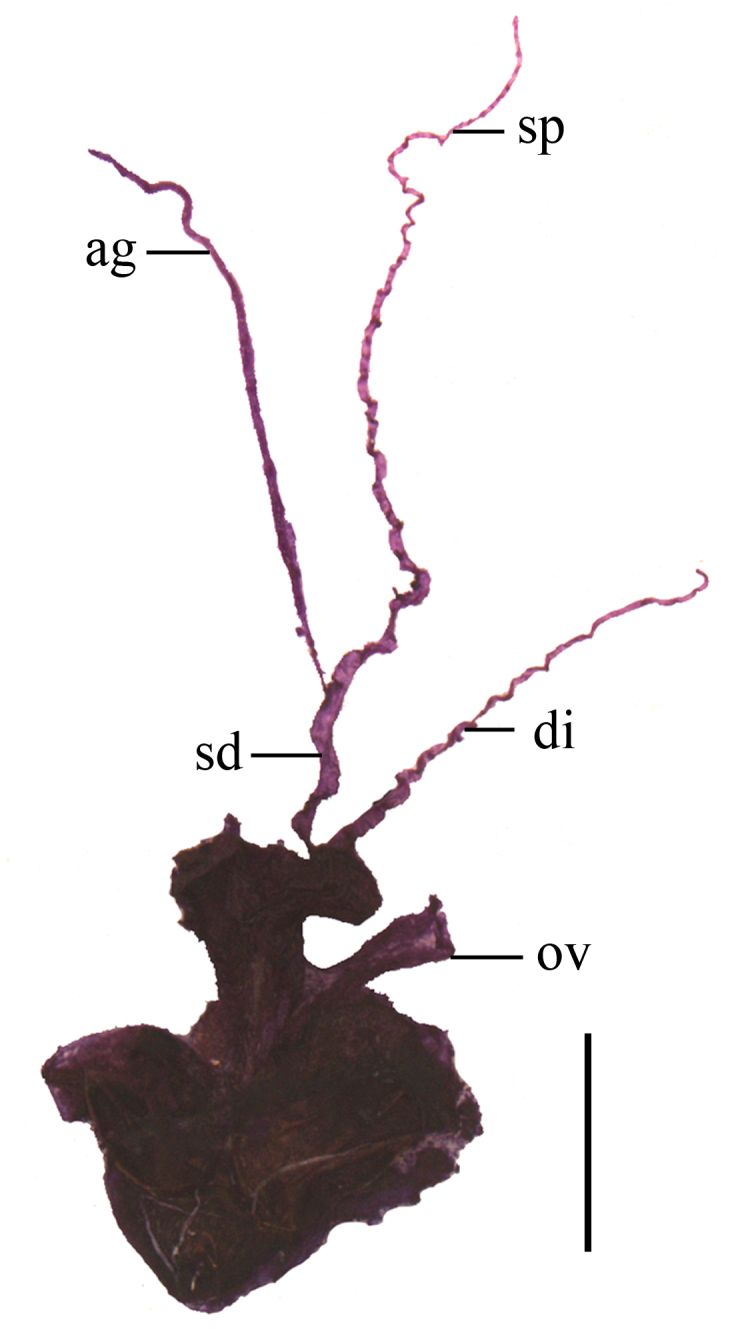
T. liangi sp. nov., internal organ of female reproductive system, lateral view.
